# Mind the gap? The platform trial as a working environment

**DOI:** 10.1186/s13063-019-3377-5

**Published:** 2019-05-29

**Authors:** Liz Morrell, Joshua Hordern, Louise Brown, Matthew R. Sydes, Claire L. Amos, Richard S. Kaplan, Mahesh K. B. Parmar, Timothy S. Maughan

**Affiliations:** 1 Health Economics Research Centre, Nuffield Department of Population Health, University of Oxford, Oxford, UK; 2 Oxford Healthcare Values Partnership, Faculty of Theology and Religion, and Harris Manchester College, University of Oxford, Oxford, UK; 3 MRC Clinical Trials Unit at UCL, Institute of Clinical Trials and Methodology, University College London, London, UK; 4 CRUK/MRC Oxford Institute for Radiation Oncology, University of Oxford, Oxford, UK; 5 Oxford University Hospital Foundation Trust, Oxford, UK

**Keywords:** Precision medicine, Stratified medicine, Biomarker, Platform trial, Trial management, Qualitative, Efficiency, Researcher, Adaptive design, Compassion

## Abstract

**Background:**

Trials have become bigger and more complicated due to the complexity introduced by biomarker stratification, and the advent of multi-arm multi-stage trials, and umbrella and basket platform designs. The trials unit at University College London has been at the forefront of this work, with ground-breaking trials such as STAMPEDE and FOCUS4. The trial management and data management teams on these trials have summarised the operational challenges, to enable the broader clinical trials community to learn from their experiences. In a small-scale qualitative study, we examined the personal experience of individual researchers working on these trials.

**Commentary:**

We found reports of high workloads, with potentially significant stress for individuals and with an impact on their career choices. We conclude that there was an initial underestimation of the work required and of the inherent, largely unanticipated, challenges. We discuss the importance of fully understanding these trials’ resource requirements, both for those writing grant applications and critically, for those with responsibility for deciding on funding.

The working environment was characterised by three features: complexity, scale and heightened expectations. These features are highly attractive for professional development and engender high levels of loyalty and commitment. We observed a trade-off between these intrinsic rewards and the continuous demands of overlapping tasks, balancing a mix of routine and high-profile work, and the changing nature of pivotal roles. Such demands present challenges for colleague relationships, by enhancing the potential for competition and by disrupting the natural opportunities to pause, review and celebrate team achievements. In addition, molecular stratification in effect brings the patient into the trial office, as a specific individual, despite anonymisation, who is owed test results and a treatment decision. We discuss these observations with a view to interconnecting the need for compassion for patients with caring for the researchers engaged in the research ecosystem who are aiming to produce much hoped-for advances in medical science.

**Conclusions:**

There is a need for increased awareness of the challenge these studies place on those throughout the team delivering the study. Such considerations must influence leaders and funders, both in their initial budget considerations and throughout delivery.

## Background

Clinical trials in the 21st century are evolving from the traditional two-arm comparison of an experimental treatment vs. a control, to accelerate identification of promising therapies, to increase throughput and to allow for the increasing use of molecular classification of patients into smaller sub-groups [1]. ‘Platform protocols’ is a broad descriptor of protocols that allow for the simultaneous evaluation of multiple treatments within at least one disease area, in some cases against a common control [2, 3]. (The term ‘master protocol’ may also be used [3]; here we use the term ‘platform protocols’ for consistency with the papers that provoked this work.) Such trial types may also be adaptive in design*—*such as the multi-arm, multi-stage platform design*—*whereby pre-planned interim analyses allow interventions with little evidence of benefit to be terminated quickly and new arms to be added as further technologies emerge [1, 4]. The adding and dropping of arms from a protocol is an important feature of the efficiency of the design, but it adds substantial complexity to the operational delivery of a study. As these approaches are relatively new, there is as yet limited research literature on the operational challenges of these more complex trial designs.

The Medical Research Council’s Clinical Trials Unit at University College London (UCL) has been at the forefront of developing and implementing such platform protocols. In the accompanying two papers, researchers share their observations on the operational challenges of such trials, to enable the broader clinical trials community to learn from their experiences. Schiavone et al. describe the experiences of the trial managers [5], while Hague et al. write from the perspective of data management [6].

In this commentary, we provide an additional perspective to their observations by exploring the human experience of the researchers working on these complex trials. In a small-scale qualitative project, we examined the effect on individuals, their lives, their careers and their job satisfaction. This work stems from the interest of three of the authors (LM, JH and TSM) in the societal significance of the precision medicine paradigm [7, 8] working with a diverse group encompassing clinicians, psychologists, patient advocates, health economists, ethicists and theologians. We have applied the broad perspective of that group in considering the implications here, drawing on a project of the Oxford Healthcare Values Partnership entitled *Compassion in healthcare: practical policy for civic life*, which considered not only the role of compassion in relationships between clinicians and patients [9] but also the care offered to colleagues and teams working in healthcare [10].

We emailed researchers at a single unit to invite participation, with no follow-up and no compulsion. We acknowledge that the individuals attracted by this light-touch recruitment approach may not be fully representative, but our main concern was to encourage an open conversation and to establish the independence of the interviewer from the unit’s management. The interviews were minimally structured. Each discussion was deliberately left open to follow the issues raised by the interviewee, rather than following a structured discussion guide with specific questions predefined by the interviewer. Altogether, 18 researchers volunteered to speak to us, resulting in over 15 h of recorded and transcribed interviews. We used the recordings, interview notes and transcripts to identify key topics. Information from the interviews was extracted into a grid under these headings, which were grouped into overarching themes.

As the discussions covered potentially sensitive topics, we committed to preserve the anonymity of all participants. For this reason, combined with the small scale of the study, we share our observations here as a summary overview. Our observations are organised under four main themes: we link to the findings in the UCL papers, and provide commentary on implications for individuals, trials units and funders.

## Efficiency

*I think I, at least, underestimated the work that was involved, to begin with.*

For the readership of *Trials*, there is no need to rehearse the expected methodological efficiencies of platform protocols. What we and the trials unit found, though, was that the central resources needed to achieve the practical efficiencies were greater than expected, when the trials expanded. Schiavone et al. describe the work involved in adding a new comparison by amending an existing protocol [5]. Hague et al. discuss the work involved in changing the database to accommodate additional comparisons without impacting the ever-growing data from ongoing comparisons [6]. Both papers note the additional work entailed in trials involving biomarker stratification.

The impact for individuals of the resulting unanticipated activities is a high workload, leading to a sense of not being in control. Importantly, there is an impact on individuals’ career choices, particularly with regard to part-time working:

*It’s just never enough.*

*This is not a job you could do part time.*

Whilst workloads were reported to have reduced as the understanding of resource needs improved, there was an inevitable lag, due to limited flexibility to increase funding mid-grant. It is, therefore, essential to improve our understanding of the resource requirements of platform protocols for those writing grant applications, but perhaps even more importantly, for those responsible for reviewing them and making funding decisions. The two UCL papers make a valuable contribution to this discussion.

For trials units and lead investigators, a keen awareness of the staffing required to meet the repeated set-up and data management activities will help to calibrate the budget setting process. Contributions from commercial partners may also be challenging to quantify, as it is very rare for contracts to be signed and for funding to be agreed prior to grant submission. Over-optimism regarding commercial contributions is a real danger, as partners are prepared to contribute free drugs and distribution costs often only until they see a clear line of sight for commercial development. These factors can be very difficult to predict, so flexibility around the funding of such treatments is likely required, which can be challenging to administer and agree.

Further, investigators and funders can benefit from engagement with patient representatives both at the proposal stage and throughout the life of the trial. The trials discussed here have active patient representatives. It may be that the questions posed to patient and public involvement representatives need some reconsideration to ensure that there is a focus on the things that really matter.

Reassuringly, issues of workload are inherently manageable once resource needs are understood. Our remaining themes characterise the nature of that work and its impact on individuals and the working environment.

## Complexity

*We’re doing all of the life cycle of a normal trial—but all within the same time point.*

Both UCL papers refer to the parallel workload of running multiple trial arms that are at different stages in the life cycle of a trial [5, 6]. Although from a trials unit perspective it is usual to have multiple trials at different stages, in a platform protocol, this breadth is more likely to be experienced by an individual. For researchers, this creates challenging and fast-paced work, and exciting career development opportunities. Within a short period, a researcher can be exposed to, and gain experience in, all stages of a trial:

*I got the experience in five years—even I guess in less than five years—of what some [researchers] wouldn’t get in ten years.*

*Anything else would be really simple. I’d maybe be a bit bored.*

However, individuals also have to manage multiple overlapping tasks and continually need to make priority choices. In particular, staff noted the conflict between day-to-day tasks and higher-profile work, like opening new arms, with limited flexibility to adjust time points for activities specified by the adaptive design:

*It’s the fact that you've got to do that in parallel with the start of new trials, and collecting data from the arms that have been running for years all at the same time.*

The continual demand from different tasks disrupts the normal peaks and troughs in work and obscures the natural points at which a team can pause, celebrate and mark progress, and reflect:

*There is never a point where you say, ‘And we're done.’ It's always: ‘OK what’s the next move?’*

To prevent these necessary breakpoints from becoming submerged and lost, trials units need to develop conscious mechanisms to identify such key points, so that teams can mark their progress and acknowledge the effort taken to achieve it.

With so many exciting career opportunities, we observed a potential for turf conflict, highlighting the importance of the clear and transparent allocation of tasks. However, it is important that trials units do not respond by narrowing roles, such as by asking individuals to focus on a particular type of task. Whilst some clarification of roles is helpful, this needs to be balanced against maintaining the challenges and career development opportunities that researchers find so stimulating in these trials:

*There’ll be one or two data managers who are just doing data entry. … That’s not necessarily a great job to have 100% of your time.*

## Scale

*I was the first ever ‘third data manager’.*

As these trials have grown, so have the trials unit teams. Schiavone et al. comment on the need for larger central teams [5]; Hague et al. discuss the impact of scale on database design and structure [6]. Both note that the expanded scale applies to longevity as well as people, and combined with the large number of trial sites, creates added demand for ongoing training:

*Say you’ve got 100 sites, and you’ve got maybe two people that can do data entry. And out of those, some of them are going to be leaving that year, … so then you’ve got to train up the next person.*

Whilst issues of scale are not unique to platform protocols, they clearly affect the nature of the work. For a trials unit, the project management challenges presented by these large trials are considerable at all levels, including senior leadership. Even able and committed chief investigators and clinical leads are unlikely to have received the training and support required to equip them for a general management role that is potentially equivalent to that of a chief executive officer of a medium-sized company.

Importantly, the expanded project scale significantly changes the role of the data manager, and perhaps even more so, the clinical trial manager, from being a central contact point with sole responsibility for all aspects of a trial to being one member of a team. This can create challenges in maintaining the motivation of individuals, who may feel that their role has been diminished and is less satisfying, or feel a loss of ownership for a trial they are working on:

*Once you’ve got more trial managers, their centrality is lost.*

In addition to supporting the current staff in adjusting to their changed role, trials units may need to revise employment criteria to ensure that new trial managers have clear expectations and a good skill fit for the new role.

## Hype

*I love that study. It’s a great trial.*

This theme relates to the effect of working on high-profile successful trials considered groundbreaking in some way. For one of the trials, part of the potential hype was the current high profile of personalised medicine, whilst for the others it was the novelty, scale and successful history of the project.

The result for individual researchers is a strong attachment to these trials, because of their novelty, the cutting-edge science, the intellectual challenge and their potential to benefit patients:

*It’s quite motivating, the fact that we are quite a flagship trial and it has changed standard of care.*

*This is the first time I’m doing something that I would say was really exciting.*

These attachments created a sense of commitment and loyalty, in a range of different ways: loyalty to patients who are waiting for biomarker results, to the history and success of the trial, and to good science through generating quality results and meeting the commitments declared in the protocols, to funders and to regulators. The sense of loyalty adds to the demands that individuals experience in working on these trials, particularly if the trial is highly visible:

*I never want to be the person who’s got it stuck with me.*

*It has to look like it’s going well.*

Notably, the biomarker-stratified trial has the effect of making staff in the trial office aware of specific patients in a unique way compared to non-stratified trials. Despite anonymization, there is, nonetheless, a sense of the trial being about specific individuals who are owed individualised test results and treatment decisions. This sense of engagement with patients was shared by the laboratory, who see first-hand how few patients are actually eligible for targeted treatment:

*We are very aware of the numbers. The numbers of patients who go out with kind of a good news report and then others who go out with a ‘Sorry. We haven’t found anything that’s going to change your treatment.’*

Within the trials unit, the hype can impact researchers working on other trials. When flagship trials received internal recognition or external publicity, some commented on the negative reactions from staff not involved with the trial and the demotivating effect on colleagues when “other great things … didn’t get as much airtime.”

*There used to be—not kind of audible groan, but whenever it was talked about some people then were like: Oh, [trial] again ….*

Overall, in addition to the work that has to be done (known as extrinsic demand in the literature on workplace stress [11]), the sense of hype surrounding these high-profile trials appeared to strengthen the intrinsic demand stemming from the motivation of the individual. This presents a challenge for the leadership of trials units in protecting the well-being of researchers on these trials without blunting their commitment, and at the same time, sustaining motivation for those working on less high-profile trials.

## Compassion and care

Schiavone et al. and Hague et al. provide practical suggestions for addressing the operational challenges of platform trials. Here we approach the findings from a different angle, that of compassion and care, which raises questions about the ethos of platform protocols. Previous work has paid attention to the interrelation between compassion and risk in the ethos of stratified medicine in general, with a particular focus on the interrelation between patients and clinicians [9], and caring for colleagues and teams working in healthcare more generally [10]. Here, the more delimited focus is on the context of a *trials unit* as a context for compassion and care.

First, the phenomenon noted above in the context of biomarker-driven trials, whereby staff in the trial office become aware of the treatment options for specific patients, (albeit anonymised), has significantly reduced the emotional distance *between patients and researchers*, making a somewhat attenuated kind of *individualised* compassion possible. Researchers feel the urgency of their *duty* to attend to a test speedily and return the results. Their work draws them into the needs of individual patients and thus, into a kind of personalised relationship. However, this relationship lacks meaningful access to the normal bases of compassion in clinician–patient relationships (e.g. the patient’s background, life journey and what is important to them at this point), which make possible a rich, interpersonal experience.

The challenge for those working in a trial setting is that the incipient compassion they may feel—albeit anonymised with no name, face or story to attach to the assay—is contextualised by the trial team’s overall awareness of the numbers of patients involved. The trial team have insight both into the lack of therapeutic options for the majority of patients and thus, the likely gravity of the consequences for many individuals. This experience regarding individual patients indicates that trial teams should consider how to support researchers over the long term if results that indicate targeted therapeutic options are relatively *infrequent* while optimism remains *high*.

The challenge is how the reduced emotional distance between bench and bedside, which lacks the face-to-face quality of clinical relationships, can nonetheless enhance the compassionate sense of purpose, which researchers may see as key to their sense of vocation. A way to address this would be to bring elements of the ongoing narratives of anonymised patients being served by the trial team—whether those patients have been eligible for targeted treatments *or not*—into team meetings, providing more of a sense of how the trial team’s work is interacting with real life stories. Twice yearly meetings at which research nurses involved in trial delivery at trial sites share such participant stories with the overall team could accomplish this and enhance the research nurses understanding of the trials unit’s needs, to the mutual benefit of both parties. This approach would be a sensitive complement to the increasingly common practice of involving patient representatives in the design stage of trials and throughout a trial’s lifetime, as noted earlier.

Second, notwithstanding that working on a trial with a platform protocol can be exciting and career-enriching, staff reports of the significant difficulties and stresses of such work call for reflection on what caring for *researchers’ well-being* might entail. In particular, the challenges of scale and complexity alongside a built-in and progressive reduction in moments for rest or celebration disrupt the opportunities for the trial to be experienced as a shared journey, a narrative that would facilitate staff members’ self-care and care for each other. Similarly, a drive for efficiency that narrows staff experience and opportunities may lead to demoralisation and fragmentation rather than collegiality. Accordingly, identifying those key points where teams can share their common achievements and disappointments should also afford opportunities for the stresses and strains on teams and individuals to be acknowledged and engaged with.

This process is particularly important because of the loyalty that trial team members have articulated towards the trials they are involved in, especially to the history of the trial and thus, the fear of its failing to achieve the hoped-for patient benefits. Caring for staff will require acknowledging the emotional costs to researchers of this level of commitment. After considering our interviewees’ overall attitudes to these trials, we found that their strong attachment and motivation conflicts with the chronically high demands on their time and energy. Such committed, loyal researchers may be less likely to consider or talk about their own well-being and therefore, less likely to access support or raise concerns. Practical issues for teams to consider include creative role management, careful engagement with line managers and human resources personnel, and career counselling. A caring ethos for such trials would require that loyalty to the shared hope in a trial’s future promise does not trump the needs of colleagues in the present.

## Conclusions

Complex, adaptive studies with the added challenge of molecular stratification are essential for driving forward a new era in clinical trials, particularly in precision medicine. They represent a form of collaborative science in which clinicians, statisticians, biomarker laboratory scientists, trial managers and data managers all play a critical role. Leading such enterprises, for both chief investigators and trials unit leads, can be demanding and time-consuming, and yet little is being done to train or prepare such individuals. Awareness of patient perspectives through end-to-end engagement with patient representatives is now expected as standard. In addition, there should also be increased awareness of the challenge such studies place on everyone in the team delivering the study. Ensuring that researchers’ connection with patient perspectives and narratives, and with each other’s experience in the trial, are vital elements of a compassionate and caring trial environment. These considerations must influence leaders and funders, both in their initial budget considerations and in the long haul of delivery for such exciting research.

## Abbreviations

UCL: University College London
